# Providing medicines-related support for people with COPD before and after hospital discharge—a qualitative study of hospital staff perspectives

**DOI:** 10.1186/s12913-025-12992-3

**Published:** 2025-07-02

**Authors:** Torbjørn Nygård, David Wright, Reidun L. S. Kjome, Hamde Nazar, Aase Raddum

**Affiliations:** 1 Centre for Pharmacy/Department of Clinical Science, University of Bergen, P.O. Box 7804, Bergen, 5020 Norway; 2 School of Healthcare, University of Leicester, Leicester, UK; 3 Centre for Pharmacy/Department of Global Public Health and Primary Care, University of Bergen, Bergen, Norway; 4 School of Pharmacy, Newcastle NIHR Patient Safety Research Collaboration, Newcastle University, Newcastle Upon Tyne, UK

**Keywords:** Health services research, Qualitative research, Focus groups, Interviews as topic, Pulmonary Disease, Chronic Obstructive, Medication therapy management

## Abstract

**Background:**

People with chronic obstructive pulmonary disease (COPD) are frequently admitted to hospital and experience challenges with their medicines. Changing service delivery to address medicines-related challenges has been shown to reduce readmissions and improve patient outcomes. Before attempting to improve medicines-related support through new interventions, it is necessary to firstly understand contextual factors surrounding the delivery of current usual care. The aim was to identify improvement areas of medicines support during and after hospital discharge, and why this support is not always provided.

**Methods:**

Hospital pulmonary ward staff were included in a focus group and semi-structured interviews. Data were analysed through systematic text condensation.

**Results:**

Six major themes were developed and classified as organisational or practitioner level. Organisational level themes were: (1) transfer between care levels is challenging, (2) follow-up lacks coordination, and (3) low financial resources. Practitioner level themes were: (4) competence about COPD is needed, (5) clarification of professional role and task distribution, and (6) practitioners need to educate and support patients.

**Conclusions:**

Medicines support for people with COPD during and after discharge would benefit from undertaking medicines reconciliation and increasing coordination across care levels. Furthermore, choice of inhaler devices should not be limited by reimbursement systems. Medicines support interventions should be adapted for primary and secondary care settings or include collaboration across care levels.

**Supplementary Information:**

The online version contains supplementary material available at 10.1186/s12913-025-12992-3.

## Background

Chronic obstructive pulmonary disease (COPD) is an irreversible and progressive disease [1]. People with COPD experience revolving door syndrome, in which people who are admitted to hospital for COPD are frequently readmitted to hospital [1]. With disease deterioration, the use of and need for medicines increases. Polypharmacy is common among people with COPD. This patient group often experience medicines-related issues [2], such as non-adherence to medicines and incorrect use of inhalers [3]. It is warranted to resolve and prevent these issues to optimise therapy and improve health outcomes for people with COPD [4, 5]. The point of hospital discharge provides an opportunity to ensure optimal use of medicines which may help to prevent readmissions.

While complex interventions such as pulmonary rehabilitation and self-management strategies have been recommended to improve patient outcomes and managing medicines, not all healthcare systems provide these interventions and participation rates are often low [6, 7]. Providing discharge care bundles is another approach, in which optimising medicines is recommended. However, evidence on effectiveness is limited and many challenges to implementation exist [1, 8]. Recent research has recommended that challenges in usual care should be addressed before implementing new interventions for COPD patients [9].

Including stakeholders is key to developing new complex interventions or making changes to existing practices, as recommended by the UK’s Medical Research Council [10]. In this study, the aim was to identify what could be improved related to medicines support during and after hospital discharge, and why this support is not already routinely provided. Therefore, we included practitioners from the hospital setting as stakeholders to enable us to increase our understanding of hospital staff perspectives and experiences of COPD medicine-support. This information will be used to optimise standard care and to inform the development of future interventions.

## Methods

### Study design

This qualitative study had a phenomenological approach with a combined use of focus group and semi-structured individual interviews. The reporting of this study was underpinned by the Consolidated Criteria for Reporting Qualitative Research (COREQ) and the checklist is provided in the Supplementary Material.

### Setting

All participants of this study are associated with the pulmonary ward at a university hospital in Norway. The pulmonary ward has two separate clinics, i.e., inpatient and outpatient. Pulmonary rehabilitation is provided by the hospital outpatient clinic. The hospital serves a large regional area consisting of multiple municipalities of various size. Each municipality controls its own budget and is responsible for providing its inhabitants with primary care services, such as general practices, nursing homes, and home-based care.

### Recruitment

Convenience sampling was used to recruit participants. Participants were eligible for inclusion if they were health care personnel working at the hospital and had experience working with people with COPD. Study information was distributed at the pulmonary ward through e-mail, additionally, an oral presentation about the study was provided for the physicians. Participants in the focus group consisted of nurses and nursing assistants working at the pulmonary inpatient ward. These participants were recruited via an administrative nurse at the ward. Participants in the individual interviews were physicians working at the inpatient or outpatient pulmonary ward. An invitation to join the study were distributed through an online form to all physicians associated with the pulmonary ward. Additional information about the study and data protection was provided in the interviews before verbal consent was collected from the participants.

### Data collection

The focus group was conducted in December 2023 and the individual interviews were done January–February 2024. Two separate interview guides were developed prior to data collection, one for the focus group and one for individual interviews (See Supplementary Material). The interview guides were discussed and revised within the research team but not pre-tested before interviews. A pre-test of interview questions would have strengthened the dialogue and comprehension of questions.

The focus group was conducted in a meeting room at the pulmonary ward where TN was the main moderator of the focus group and RK was co-moderator. The individual interviews were conducted in available offices/meeting rooms at the hospital. TN conducted the interviews. The focus group and the individual interviews were audio recorded, and short written notes were made during the interviews and focus group. The interviews were manually transcribed by TN.

The interviewers had no previous relationship with participants. However, occupation (i.e., pharmacists in research) and goals for undertaking the research was shared with participants in the beginning of the interviews/focus group. Moreover, early findings from a systematic review [9] and a patient interview study [11], previously undertaken by the research team, were shared with participants to build rapport and to introduce the research problem with examples.

Information power was used by the author team to guide sample size estimation and the recruitment strategy [12, 13]. The aim of the study was narrow (i.e., exploring medicines-support provided for people with COPD in the hospital/post-discharge setting), the sample specificity was dense (i.e., clinical healthcare professionals with expertise in COPD), the study was informed by theory (i.e., systematic review [9] and patient interview study [11]), and the dialogue was strong (i.e., participants and researchers are healthcare professionals with common knowledge about patients with COPD). A cross-case analysis was undertaken (i.e., common themes derived from multiple participants/professions), which would require more participants. Overall, the resulting information power of this study was high—especially due to including participants with high expertise—and therefore required fewer participants.

### Analysis

An inductive analysis was undertaken using systematic text condensation developed by Malterud [14]. The analysis was undertaken in four steps: (1) familiarisation with the data through reading the transcripts multiple times and constructing preliminary themes; (2) identification of meaning units (i.e., codes) in the data and reconstruction of themes; (3) condensation—creating subgroups within the themes, decontextualisation, and creating artificial quotations; (4) synthesising by recontextualisation and creating analytic texts for each subgroup of themes. Each step of the analysis was undertaken by multiple researchers to facilitate broader interpretations. An idiomatic translation from Norwegian to English was undertaken in the second step of the analysis when identifying meaning units. The coding process was undertaken using MAXQDA 2024 (VERBI software).

## Results

One focus group interview and three semi-structured individual interviews were conducted. The focus group included four nurses and one nursing assistant. The nursing assistant is reported as a nurse throughout this study. The individual interviews included three physicians, two pulmonologists and one physician undergoing final specialisation in pulmonology. The focus group lasted 60 min, and each individual interview lasted 30–50 min. The dialogue in the focus group and interviews was strong and provided in-depth information about the studied phenomena.

**Fig. 1:**
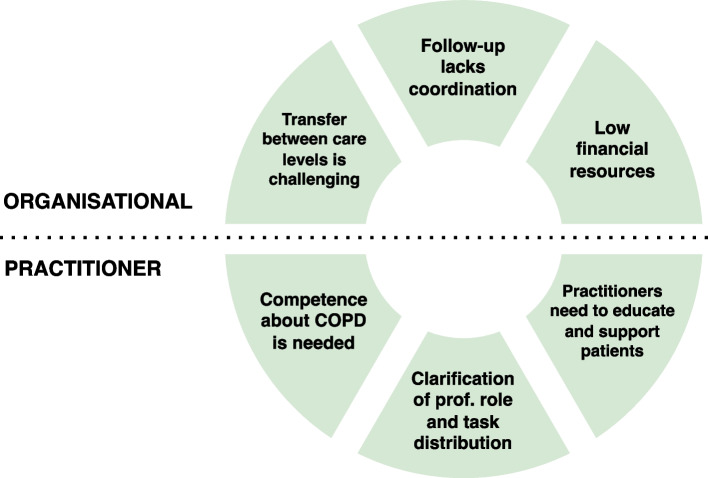
Major themes from the analysis at organisational and practitioner level. Prof.: professional

Six major themes were constructed and separated into organisational level and practitioner level (Fig. 1). Minor themes were also developed at the patient level, these are reported in the Supplementary Material, and not included in further analysis.

### Organisational level

#### Transfer between care levels is challenging

The hospital staff experienced difficulties with different municipalities when they organised transfer from secondary to primary care. It was difficult for the pulmonary ward staff to have an overview of which healthcare services were available for the patients in the different municipalities. Furthermore, they knew little about how their recommendations were followed up in primary care between hospitalisations. The hospital nurses reported that local care coordinators in primary care sometimes lacked understanding about the increased care needs the patients have following hospitalisations, specifically if the patient was already enrolled in the primary care system. In these cases, the care coordinators would assume the patient could return to their original arrangement after discharge from hospital.

*[…] some municipalities are more difficult than others, […] it’s almost like we can predict [outcomes] based on which municipality it is. […] they don’t always want to listen to us, […] so it’s very important that we specify the increased needs for help [following hospitalisation]. (Nurse 4)*

Medicine lists were often not updated by hospital physicians. Medicine reconciliation was not always done due to time constraints, despite it being mandated by law. This sometimes resulted in medication errors, such as concurrent use of similar therapies (i.e., therapeutic duplication). Furthermore, lack of interoperability between electronic health record (EHR) systems across care settings created more practical issues with updating medicine lists. The hospital staff suspected that poor communication between prescribers and with patients was a major factor contributing towards this issue.

*I sometimes have patients who are on Symbicort (budesonide/formoterol), Trimbow (beclomethasone/formoterol/glycopyrroniumbromide), and Spiriva (tiotropiumbromide) at the same time, so they are essentially on double triple therapy because the patient hasn’t quite understood that these medications actually work in the same way. The general practitioner finds it too complicated to go through all the medication lists, and because they don’t ask what each patient is using. I think communication is a big part of the issue. (Physician 3)*

#### Follow-up lacks coordination

While patient education on medicine use is provided in the outpatient clinic, pulmonary rehabilitation, surgeries or pharmacies, the overall responsibility for follow-up is not clearly delegated after patients are discharged. The hospital staff experienced that there was limited time for follow-up for each patient in the outpatient clinic. They saw hospital-provided pulmonary rehabilitation as an appropriate setting to provide information on medicines, but the education and training lacked individualisation. The pulmonary ward staff were aware of other pulmonary rehabilitation services—which they did not provide themselves—but had limited information about these services and assumed that patients had the same issue.

*No, it’s difficult because the services in the municipality vary from time to time, and it varies from municipality to municipality, so what is available in the different municipalities is very hard for us to keep track of, even what exists in [our home municipality]. The surrounding municipalities are also incredibly difficult to know, but it would probably be possible to create some sort of overview, mhm. But that information doesn’t seem easily accessible to me. What I would like is for us to have an overview, but right, someone would have to update it, but I don’t think anyone has that as a task. (Physician 1)*

The hospital lacks a proper communication channel which could allow primary care to take advantage of the competence among the pulmonary hospital staff. They contrast this to patients with cancer, who have nurse coordinators in the municipality who help them to navigate and coordinate healthcare services.

*[Primary care healthcare professionals] must be able to utilise the expertise that is in hospitals to a greater extent, for training, to ask for help when they have issues, they need a channel into the hospitals. […] But maybe selected patients could have a nurse or a team in the municipality or at the hospital they could contact if there were things that were difficult. (Physician 3)*

#### Low financial resources

In primary care, it varies greatly how much resources each municipality have for rehabilitation. Therefore, the hospital staff found it difficult to know what awaits the patients when they are discharged, because patients may or may not receive sufficient rehabilitation and it is not standardised.

The hospital staff were aware that it is crucial for people with COPD that healthcare services they receive are fully financed, as many in this patient group have a low socioeconomic status. Even modest out-of-pocket payments may be a barrier to care. In Norway, costs for vital medicines are shared between out-of-pocket payments and state reimbursement. Medicines are fully reimbursed when patients reach a set limit for out-of-pocket payments. This is not optimal, because some patients struggle at the beginning of each year to pay for their medicines, and the staff told of patients reporting that they had to prioritise which medications to buy. Furthermore, while the participants recognise that the fully reimbursable non-proprietary medicines may save money for the healthcare system, they had experienced that changing to non-proprietary inhalers often caused confusion in patients due to different instructions for use. This could lead to incorrect use and potentially cause costly hospitalisations.

*[Pulmonologists], as a professional community, protested against the introduction of non-proprietary substitutions for inhalers, precisely because we spend a long time training [the patients], and the patients take a long time to get used to the medicine, and then it suddenly gets turned on its head. Which I strongly disagree with. I still haven’t heard a good argument other than saving NOK 30–40 here and there. Which I don’t think is worth it. It doesn’t take many exacerbations of asthma or COPD before all the money [saved on substitutes] is used up, I think. (Physician 3)*

### Practitioner level

#### Competence about COPD is needed

The pulmonary ward staff experienced that there is a lack of knowledge about inhalers and medical devices used in COPD in non-specialised healthcare institutions, including other hospital wards, intermediate care units, and home care services. The hospital nurses believed that nurses in home care services have insufficient knowledge about correct use of inhalers and other medical devices. They had experienced that patients needed longer stays in hospital when the municipality lacked the competence to care for the patients: *“So they probably need an extra day in hospital because [home care services] do not have the competence, when [the patients] really could have been home,”* (Nurse 4). However, they believed that the lack of knowledge in home care services could be resolved by hospital staff or pharmacists providing training for home care staff.

#### Clarification of professional roles and task distribution

The hospital physicians communicate electronically with general practitioners about medicines but experience that general practitioners hesitate to make changes to the medicines that are initiated in hospital. Hospital staff lack knowledge about the organisation of pharmacies and how their EHR-systems work. They believe that pharmacists can and should detect errors in prescribing, and that it would improve treatment quality if pharmacists would undertake medicines reconciliation. Additionally, yearly medicine reviews could easily be done by pharmacists according to one of the physicians. The hospital staff also believed it would be beneficial if vaccination status were checked in pharmacies. They see the inhaler training service that is provided in pharmacies as good support, as time is limited in the hospital—especially in the outpatient clinic.

When hearing about how pharmacies in the UK can offer non-prescription rescue packs (which includes steroids and antibiotics), the hospital staff believed rescue packs could be beneficial if follow-up with physicians were included. However, they were worried that rescue packs could facilitate overuse of medicines, as they believe patient demand for this would be high.

[when asked about rescue packs from pharmacies] I imagine many would have done it because they have a lot of anxiety and such, so they would have done it immediately when *imitates gasping for air*. (Nurse 2)

#### Practitioners need to educate and support patients

The hospital staff believes that healthcare personnel should invest more time in inhaler training with patients. The nurses at the pulmonary ward try to observe patients when they are using their inhalers and would also show next of kin how to use the devices to add a safety net for the patients. One physician pointed out that a lot could be done if patients were given structured discharge consultations, but this was often deprioritised in a busy workday where the newly admitted and sickest patients needed more attention.

*The way we work in hospitals today is not, in my opinion, facilitated towards having a good discharge conversation, and we often fail at that. And I often fail at that myself. I think we become so focused on doing the job for those who are most ill and admitted, that discharging them often becomes a rushed task. A lot of things are done too quickly. And I think we could have had a structured or standardised way to follow up with patients, what we could offer them after discharge. (Physician 3)*

The same physician also believed that patients should be followed up a few weeks after discharge, and that check-lists could easily support healthcare providers in primary healthcare in doing so. One of the physicians usually advised patients to receive inhaler instructions in the pharmacy and recommended that they watch videos online. The hospital nurses believed the hospital pharmacy to be good at providing inhaler training but had the impression that the training was often rushed and experienced that instructions had to be repeated in the ward. The nurses suggested that inhaler training was more suitable in a quiet room than in a busy pharmacy.

## Discussion

In this study, we have identified several areas during and after hospital discharge in which healthcare providers experience challenges with providing medicines-related support.

Healthcare providers experienced challenges when transferring patients across care levels. Regarding medicines, medicine lists were not always updated by medicine reconciliation due to time constraints. It is formally the responsibility of general practitioners to keep medicine lists updated, however, hospital physicians may need to manually inform general practitioners of any changes made to medicines. Pharmacist-conducted medicine reconciliation may help to ensure that medicine lists are updated upon transfer between care levels. Although evidence is inconclusive on the effectiveness of pharmacist-conducted medicine reconciliation on readmission rates [15, 16], undertaking medicine reconciliation would help to detect prescribing errors and ensure that an updated medicine list would follow patients across care levels [17].

Our findings suggest that the follow-up is not sufficiently coordinated across care levels. The pulmonary ward provides individual outpatient follow-up and group pulmonary rehabilitation. However, medicine support is limited in individual follow-up due to time constraints and in pulmonary rehabilitation because of a lack of individualisation. Furthermore, it is unclear who is responsible for follow-up and there is lack of resources to provide rehabilitation in primary care. The hospital staff are aware of some healthcare services in primary care, such as inhalation technique assessment service in pharmacies, but lack necessary information about the services to guide patients to access them. An integrated disease management intervention, which included a coordinator/facilitator in the primary care setting, reported positive effects on long-term hospitalisations in patients with COPD [18]. Providing patients with a coordinator to help them navigate healthcare services across care levels, as is already provided for patients with cancer in Norway, could potentially limit the fragmentation of service provision and facilitate holistic care [19].

The provision of non-proprietary inhalers in pharmacies ensures that a fully reimbursable alternative is available for the patients, however, this also increases the potential for incorrect inhaler use and patient confusion. The Global Initiative for Chronic Obstructive Lung Disease (GOLD) recommends that device types are only changed upon clinical justification [1]. Costs should not primarily determine which inhaler device is dispensed, especially because COPD is associated with low socioeconomic status [20]. More research is required to investigate the clinical impact of inhaler reimbursement systems and how to limit any negative consequences.

The hospital staff emphasise the need to increase healthcare provider competence about COPD and related pharmacological therapies. The nurses suggested that education could be provided by the pulmonary ward or pharmacists. Alternatively, digital educational programmes may be more feasible as time and resources are already limited throughout health systems [21]. It is unclear if educational interventions aimed at healthcare providers improve COPD management, however, it may improve influenza vaccination and patient satisfaction with care [22].

A qualitative study from Sweden on healthcare professional perspectives on COPD care reported similar themes to ours [23]. In particular, the authors reported that there is a lack of COPD-related competence among healthcare providers, interprofessional collaboration is lacking, and communication with the county is insufficient [23]. A larger international qualitative study explored clinician perspectives on barriers to optimal COPD care [24]. Similar to our findings, common barriers among the different healthcare systems included coordination challenges, fragmented healthcare systems, and overloaded healthcare systems [24]. Reimbursement and insurance challenges were also reported—particularly for upper-middle income countries—however, our findings on reimbursement challenges related to non-proprietary medicines were different from the other challenges reported. The consequences of the Norwegian reimbursement system on inhaler-use requires further research.

### Strengths and limitations

We used convenience sampling in this study. Although this is not the ideal sampling method, the information power was high. All participants were experienced with people with COPD in the hospital setting and had high expertise in this field. This, together with the narrow aim of the study and strong interview conversations, provided sufficient information to answer the study objective [12]. Because few participants agreed to participate, reasons for non-participation should have been collected to allow for improvements in the recruitment strategy.

Only one hospital was included in this study. Even though this is a large hospital serving a wide and varied population, as well as multiple and differing municipalities, including additional hospitals from other geographical regions would have improved credibility of findings and increased transferability to other contexts. Furthermore, the primary care setting should be explored in future research, as perspectives of community pharmacists, general practitioners, and nursing staff would provide further insight into the research problem.

Researcher triangulation was undertaken by including several researchers in the analyses, from different countries and with different areas of expertise, which provided a broader interpretation of findings. However, the research team only consisted of pharmacists and the inclusion of other stakeholders or patient representatives was limited. Furthermore, as the interviewer was a pharmacist, participants could have been influenced to provide more positive answers regarding pharmacists.

## Conclusion

Medicine reconciliation should be undertaken to ensure that updated medicine lists are used across care levels. Fragmentation of medicine support services may be reduced by effectively coordinating care post-discharge. The choice of inhaler devices should not be primarily decided by costs as this may negatively impact inhaler education and training. There is a need for increased competence about COPD in non-specialised healthcare institutions and the hospital may provide suitable training for other healthcare professionals. Medicine support interventions should be adapted for primary and secondary care or include collaboration across care levels.

## Supplementary Information

Supplementary Material 1.

## Data Availability

Data are available from the corresponding author upon reasonable request.
